# Nucleic acid visualization assay for Middle East Respiratory Syndrome Coronavirus (MERS-CoV) by targeting the UpE and N gene

**DOI:** 10.1371/journal.pntd.0009227

**Published:** 2021-03-01

**Authors:** Pei Huang, Hongli Jin, Yongkun Zhao, Entao Li, Feihu Yan, Hang Chi, Qi Wang, Qiuxue Han, Ruo Mo, Yumeng Song, Jinhao Bi, Cuicui Jiao, Wujian Li, Hongbin He, Hongmei Wang, Aimin Ma, Na Feng, Jianzhong Wang, Tiecheng Wang, Songtao Yang, Yuwei Gao, Xianzhu Xia, Hualei Wang

**Affiliations:** 1 College of Animal Science and Technology, Jilin Agricultural University, Changchun, China; 2 Key Laboratory of Jilin Province for Zoonosis Prevention and Control, Institute of Military Veterinary, Academy of Military Medical Sciences, Changchun, China; 3 Key Laboratory of Zoonosis Research, Ministry of Education, College of Veterinary Medicine, Jilin University, Changchun, China; 4 College of Veterinary Medicine, South China Agricultural University, Guangzhou, China; 5 College of Animal Science and Technology, Shihezi University, Shihezi, China; 6 Institute of Laboratory Animal Sciences, Chinese Academy of Medical Sciences and Peking Union Medical College, Beijing, China; 7 College of Life Sciences, Shandong Normal University, Jinan, China; 8 Changchun Medical College, Changchun, China; The University of Hong Kong, HONG KONG

## Abstract

Since its first emergence in 2012, cases of infection with Middle East respiratory syndrome coronavirus (MERS-CoV) have continued to occur. At the end of January 2020, 2519 laboratory confirmed cases with a case-fatality rate of 34.3% have been reported. Approximately 84% of human cases have been reported in the tropical region of Saudi Arabia. The emergence of MERS-CoV has highlighted need for a rapid and accurate assay to triage patients with a suspected infection in a timely manner because of the lack of an approved vaccine or an effective treatment for MERS-CoV to prevent and control potential outbreaks. In this study, we present two rapid and visual nucleic acid assays that target the MERS-CoV *UpE* and *N* genes as a panel that combines reverse transcription recombinase polymerase amplification with a closed vertical flow visualization strip (RT-RPA-VF). This test panel was designed to improve the diagnostic accuracy through dual-target screening after referencing laboratory testing guidance for MERS-CoV. The limit of detection was 1.2×10^1^ copies/μl viral RNA for the *UpE* assay and 1.2 copies/μl viral RNA for the *N* assay, with almost consistent with the sensitivity of the RT-qPCR assays. The two assays exhibited no cross-reactivity with multiple CoVs, including the bat severe acute respiratory syndrome related coronavirus (SARSr-CoV), the bat coronavirus HKU4, and the human coronaviruses 229E, OC43, HKU1 and severe acute respiratory syndrome coronavirus 2 (SARS-CoV-2). Furthermore, the panel does not require sophisticated equipment and provides rapid detection within 30 min. This panel displays good sensitivity and specificity and may be useful to rapidly detect MERS-CoV early during an outbreak and for disease surveillance.

## Author summary

Symptoms of MERS are atypical, including fever, cough, shortness of breath and occasionally pneumonia and gastrointestinal symptoms. As a result, it is difficult to distinguish from other respiratory pathogens based on the clinical symptoms. MERS-CoV is routinely diagnosed using real-time RT-PCR. Real-time RT-PCR has great advantages in sensitivity and specificity compared with other molecular techniques; nevertheless, it is only suitable for well-equipped or central laboratories due to the demand for sophisticated alternating temperature and fluorescent capture instruments. Here, we developed and evaluated the MERS-CoV RT-RPA-VF assay. Compared to the gold standard real-time RT-PCR, the former was less time consuming and had lower equipment requirements. Thus, this panel may be preferred to rapidly detect MERS-CoV in low-resource settings, even as a point-of-care approach for the timely prevention and control of a pandemic.

## Introduction

Coronaviruses (CoVs) are a large family of viruses with an envelope and positive-sense RNA genome that cause diseases in a broad range of hosts, including humans, cattle, swine, horses, camels, cats, dogs, rodents, birds, bats, rabbits, ferrets, alpaca, and various wildlife species [1,2]. They are classified into the genera *Alphacoronavirus*, *Betacoronavirus*, *Gammacoronavirus*, and *Deltacoronavirus*. Some members of *Alphacoronavirus* and *Betacoronavirus* can infect humans, e.g., human coronaviruses (hCoVs) 229E, NL63, OC43, and HKU1, severe acute respiratory syndrome (SARS)-CoV, Middle East respiratory syndrome (MERS)-CoV and SARS-CoV-2 [3,4]. HCoVs did not receive much attention from humans after their initial discovery in the 1960s until the outbreaks of SARS-CoV, MERS-CoV and SARS-CoV-2.

MERS-CoV has been classified as a lineage C betacoronavirus and has a genome length of 30.1 kb, which is very similar to the genome sequences of the *Tylonycteris* bat coronavirus HKU4 and the *Pipistrellus* bat coronavirus HKU5 [5,6]. Unlike SARS-CoV, which was brought under control within one year after the outbreak, MERS continues to infect people. At the end of January 2020, more than 2519 laboratory-confirmed cases have been identified in 27 countries, and more than 866 deaths associated with MERS-CoV infection have been reported since its outbreak in 2012 [7]. The majority of these cases were reported in Saudi Arabia (2121 cases), including 788 related deaths with a case-fatality rate of 37.1%. A risk of another outbreak exists after largest outbreak occurred in the Republic of Korea, Saudi Arabia and the United Arab Emirates because MERS-CoV can be transmitted by two main routes: dromedary camels-to-humans and human-to-human [8,9]. Currently, no licensed vaccines or effective therapies are available for MERS-CoV. Therefore, the rapid diagnosis of MERS-CoV is the key to successful containment and prevention of its spread.

The diagnostic method for MERS-CoV is mainly nucleic acid detection. The World Health Organization (WHO) recommends real-time RT-PCR targeting the sequence upstream of the envelope protein gene (*UpE*) and the open reading frame 1ab (*ORF1ab*) or the gene encoding the nucleocapsid protein gene (*N*) as the screening and diagnostic targets for MERS-CoV [10–12]. Real-time RT-PCR has great advantages in terms of high sensitivity and technological maturity; however, the need for sophisticated instruments has led to the delivery of specimens from low-resource settings to highly equipped centralized laboratories for testing. This approach results in a significant delay in the reporting of test results, which delays epidemic outbreak control and treatment.

Recombinase polymerase amplification (RPA) is a novel isothermal amplification technology that simulates gene recombination in phage and was established in 2006. Unlike the commonly used PCR technology, it replaces high-temperature denaturing and annealing extension in the PCR process with recombinant enzymes, single-stranded binding proteins (SSB), and strand-displacing DNA polymerase to achieves exponential amplification [13,14].

In this study, we present two nucleic acid visualization assays targeting the MERS-CoV *UpE* and *N* genes, which are rapid and highly sensitive methods to amplify MERS-CoV RNA by reverse transcription RPA (RT-RPA). In addition, a closed vertical flow visualization strip (VF) is a terminal detection device for detecting RT-RPA products, and this design is particularly applicable in low-resource settings. Compared with PCR technology, the RT-RPA-VF assay has eliminated the need for precision temperature cycling instruments [15]. Additionally, it has the advantages of being simple to implement and requires less time. Based on these properties, the RT-RPA-VF assay has been used as an alternative to the RT-PCR or RT-qPCR to detect various pathogens [16–19]. In particular, the rapid and accurate RT-RPA-VF assay has potential to reduce the risk of contagion in the early stages of an epidemic [19,20].

## Methods

### Ethics statement

Written informed consent was obtained from all the subjects prior to their participation. All swab samples that were used in the study were collected at the Institute of Military Veterinary Medicine in accordance with the approved guidelines. The Ethics Committee and Institutional Review Board of Use Committee of the Chinese People’s Liberation Army (No. SYXK2009-045) approved all the experiments.

### The rationale for the RT-RPA-VF assay

**Fig 1:**
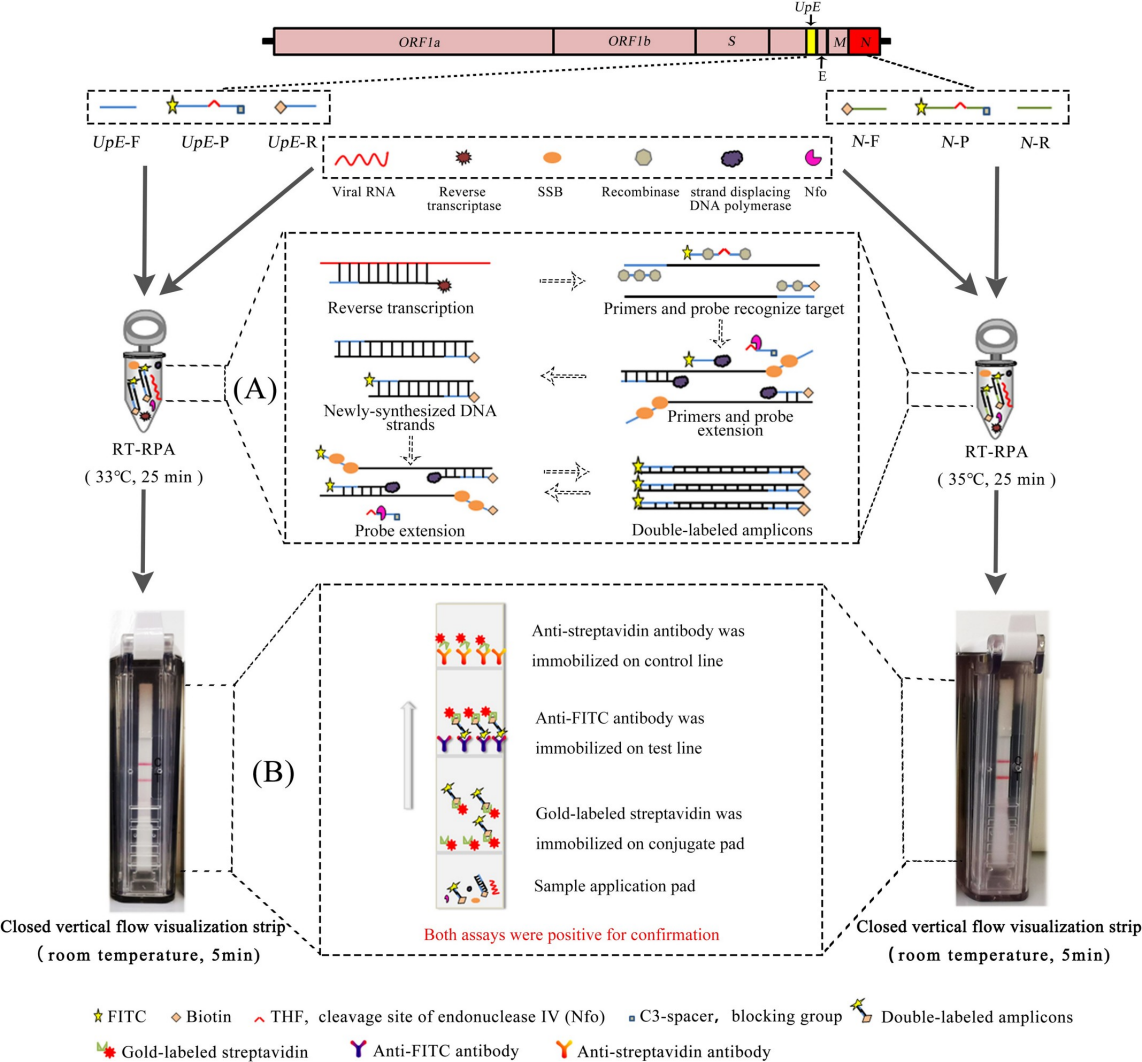
Schematic illustrating the two RT-RPT-VF assays targeting the MERS-CoV *UpE* and *N* genes. (A) The RT-RPA-VF assay was performed at a constant temperature. First, viral RNA is transcribed into cDNA by transcriptase. Second, the recombinase binds to primers/probe to form complexes that scan the template for recombination with cognate sites. Next, the strand-displacing DNA polymerase triggers primer extension events. At the same time, an SSB binds to the non-template strand to prevent the dissociation of the primer. Another enzyme, Nfo, cleaves the THF site when the probe hybridizes to its target sequence, resulting in the loss of the C3-spacer and probe extension. (C) The tube was placed in a closed device of vertical flow visualization strip device to detect the RT-RPA products. The gold-labeled anti-FITC antibody and streptavidin fixed on the test paper will capture the amplicon labeled with FITC and biotin at both ends and accumulate to form a red band.

An RT-RPA-VF assay for detecting MERS-CoV was developed by combining isothermal amplification and an immunochromatography assay. First, specific primers and probes were designed; the probe was labeled with fluorescein isothiocyanate (FITC) at the 5′ end and it contained a tetrahydrofuran (THF) residue that replaced a nucleotide and a blocking group C3-spacer at the 3′ end. In addition, the reverse amplification primer was labeled with biotin at the 5′ end. After reverse transcription, recombinant enzymes, SSB and strand-displacing DNA polymerase trigger primers extension events to synthesize double-stranded DNA. Subsequently, the probe annealed to the single-stranded target sequence within the amplification product through two primer extension. Meanwhile, the THF site of probe was cleaved by endonuclease IV (Nfo), thereby removing the blocking group and forming a new 3’ end that can be extended [21]. Finally, the amplicons were indirectly labeled with FITC and biotin due to the extension of the probe and reverse amplification primer [22]. Then, the tube containing amplicons was inserted into a closed vertical flow visualization strip device (Ustar Biotech, Co., Ltd., Hangzhou, China) and cut by a blade in the device. As liquid flows on the strip, amplicons labeled with biotin can bind to gold particles to form a complex because biotin can bind to streptavidin. When the complexes pass over the test line, they are captured by an anti-FITC antibody that is fixed on the test line, and aggregated gold particles are visualized as a red band [22]. If the test samples are positive for the target *UpE or N* gene, a colored test line appears on the strip. In contrast, negative specimens should not generate a signal at the test line (Fig 1).

### RNA and DNA templates

Total RNA extracted from hepatocellular carcinoma cells (Huh7) infected with MERS-CoV strain GD01 at a multiplicity of infection (MOI) of 0.1 and viral titer of 5×10^6^ plaque forming units (PFU)/ml was kindly provided by Professor Jincun Zhao from the State Key Laboratory of Respiratory Disease, Guangzhou Institute of Respiratory Health. Total RNA extracted from two intestinal tissue samples from bats infected with bat SARS related coronaviruses (SARSr-CoV JTMC15 strain) and HKU4 were provided by Professor Changchun Tu from the Institute of Military Veterinary Medicine. The RNA of SARS-CoV-2 was extracted from the BJ strain of the virus stored at the Institute of Military Veterinary Medicine.

The total nucleic acids of multiple respiratory pathogens were purified using the TIANamp Virus DNA/RNA Kit (TIANGEN Company, Beijing, China) from the NATtrolsp RP Multimarker Controls kit consisting of the RP Multimarker 1 (RP1) and RP Multimarker 2 (RP2) controls (ZeptoMetrix Corporation, Franklin, United States), which included 229E, OC43, NL63, HKU1, influenza A/B, rhinovirus, adenovirus, and parainfluenza, etc. (Table 1). Finally, the total nucleic acids of multiple respiratory pathogens were eluted with 50 μl of diethyl phosphorocyanidate (DEPC)-treated water.

**Table 1:** Respiratory pathogens included in the NATtrolsp RP Multimarker Controls kit.

(a)		(b)	
RP1 Respiratory virus	Strain	RP2 Respiratory virus	Strain
Influenza A H3N2	Brisbane/10/07	Influenza A H1	New Caledonia/20/99
Influenza A H1N1	NY/02/2009	Influenza B	Florida/02/06
Rhinovirus	Type 1A	RSV	Type A
Adenovirus	Type 3	Parainfluenza	Type 2
Parainfluenza	Type 1	Parainfluenza	Type 3
Parainfluenza	Type 4	Coronavirus	HKU-1 (recombinant)
Metapneumovirus	Peru 6–2003	Coronavirus	OC43
*C*. *pneumoniae*	CWL-029	Coronavirus	NL63
*M*. *pneumoniae*	M129	Coronavirus	229E
*Coxsackievirus*	Type A1	*Bordetella pertussis*	A639

(a) RP1 controls kit; (b) RP2 controls kit.

RNA transcripts of the partial *UpE* (27240–27589) and *N* (29185–29515) genes of the MERS-CoV EMC strain (GenBank No. JX869059) were synthesized by Bao Biological, Co., Ltd. (Dalian, China) at a concentration of 2.6×10^11^ copies/μl.

Six throat swabs collected from 6 healthy people and 24 oral and nasal swabs collected from Camelidae (6 healthy camels and 6 healthy alpacas) were stored in our laboratory. All swab samples were purified using a TIANamp Virus RNA Kit (TIANGEN Company, Beijing, China) according to the manufacturer’s instructions and eluted with 50 μl of DEPC-treated water.

### Synthesis of primers and probes

**Fig 2:**
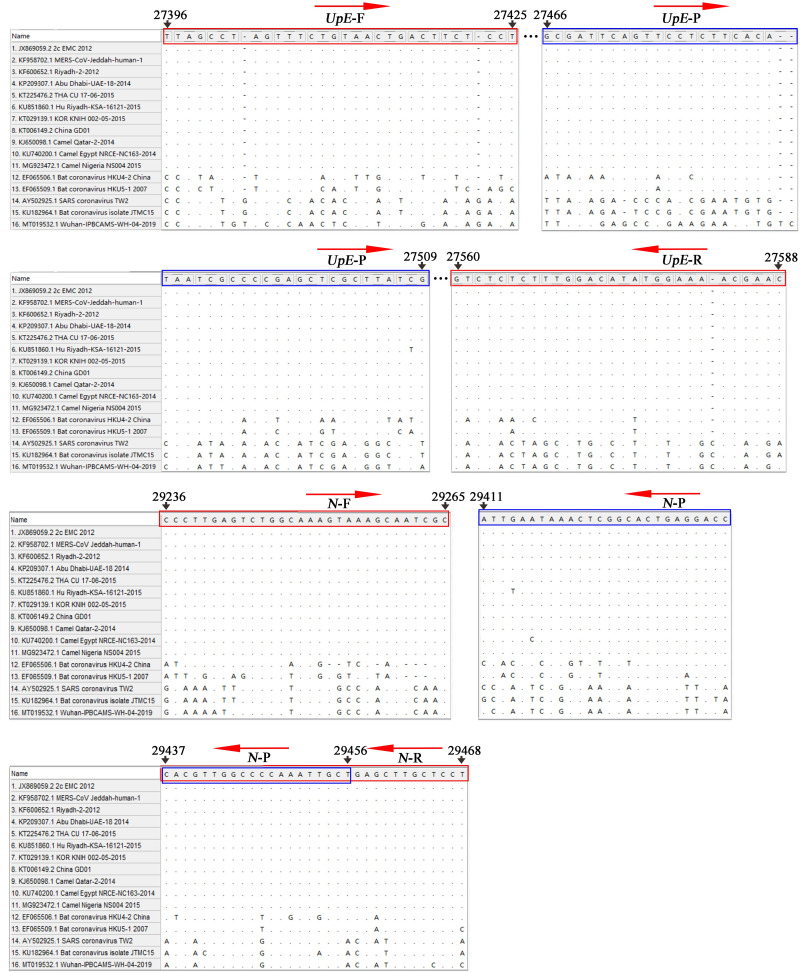
Specificity of the primers and probes used in the RT-RPA-VF assays for the MERS-CoV *UpE* and *N* genes. The sequences of genes from MERS-CoV and other CoVs were retrieved from GenBank and aligned using MEGA7 software. Only the target sequences of RT-RPA-VF assays are shown.

The complete sequences of 11 representative MERS-CoV strains from GenBank reported from 2012 to 2015 were aligned, and the conserved sequences of the *UpE* and *N* genes were screened as targets. The bat coronaviruses SARSr-CoV, HKU4, and HKU5 and the human coronaviruses SARS-CoV and SARS-CoV-2, which show high sequence homology with MERS-CoV, were also aligned to ensure primer specificity (Fig 2). The conserved sequences of the *UpE* and *N* genes of the EMC strain were subjected to an NCBI Primer-BLAST search prior to the design of primers and probes [23]. All primers and probes were synthesized by Bao Biological, Co., Ltd. (Dalian, China).

### RT-RPA-VF assay conditions and analysis of the results

According to the manufacturer’s instructions for the TwistAmp Nfo reagents (TwistDx, Ltd., Cambridge, United Kingdom), a premixed solution was prepared containing 29.5 μl of rehydration buffer, 10.7 μl of DEPC-treated water, 2.1 μl of primers (10 μM each primer), 0.6 μl of 10 μM probe, 2 μl of the template, and 0.5 μl containing 200 U of MLV reverse transcriptase (Invitrogen, Shanghai, China). The premixed solution was mixed and added to reaction tube with a freeze-dried powder containing the three enzymes needed for RPA amplification. Then, 2.5 μl of 280 mM MgCl_2_ were added to the tube cap. After mixing and centrifugation, the tubes were placed in a small metal bath at 33°C (*UpE* assay) or 35°C (*N* assay) for 25 min for amplification. A single reaction tube was transferred to the slot of the closed VF device, and then the lid of the closed VF device was squeezed to cut the reaction tube with the blade in the device, causing the reaction solution to flow into the VF. The results were observed through a closed and disposable VF at room temperature within 5 min. Three independent experiments and three parallels in each experiment were analyzed.

### Sensitivity and specificity testing

The *UpE* RNA transcript and *N* RNA transcript were subjected to 10-fold serial dilutions. Next, the RNA transcripts (ranging from 2.6 × 10^−1^ to 2.6 × 10^6^ copies/μl) were used as templates for the RT-RPA-VF assay and amplified at a constant temperature of 33°C or 35°C for 25 min. The RNA transcripts were tested with each assay in 24 replicates.

Ten-fold dilutions of the viral RNA (ranging from 1.2 × 10^−1^ to 1.2 × 10^6^ copies/μl) were also used to evaluate the two RT-RPA-VF assays under the same reaction conditions. Three replicates were analyzed in each trial.

Specificity testing was conducted in a Biosafety Level 2 facility to ensure adequate biosafety in the laboratory. The specificity of the RT-RPA-VF assay was evaluated by measuring the RNA or DNA from the SARSr-CoV, HKU4, SARS-CoV-2, RP1 controls and RP2 controls kits. The nucleic acids were used as a template for amplification under the optimum conditions of the RT-RPA-VF assay. At the same time, the MERS-CoV RNA was measured as a positive control. Three replicates were analyzed in each trial.

### RT-qPCR assay conditions

Two RT-qPCR methods for *UpE* and *N2* (Table 2) were performed using the method reported by Corman and Lu [11,12]. According to the manufacturer’s instructions for TransScript Probe One-Step qRT-PCR SuperMix (TransGen Biotech, Beijing, China), each 20 μl reaction mixture contained 10 μl of SuperMix, 6.7 μl of DEPC-treated water, 0.4 μl of 10 μM primers, 0.1 μl of 10 μM probe, 0.4 μl of the Enzyme Mix, and 2 μl of the template. The amplification reaction was performed using an Mx3005P real-time PCR instrument (Agilent Company, California, USA), and thermal cycling was performed at 45°C for 5 min, followed by 94°C for 30 s and 40 cycles of 94°C for 5 s and 60°C for 30 s. Each group included a negative control.

**Table 2:** Primer and probe sequences for the MERS-CoV RT-qPCR assay.

Genomic target	Primer or probe	Genome location	Primer or probe
***UpE***^**a**^	Forward primer	27458–27475	GCAACGCGCGATTCAGTT
	Reverse primer	27549–27530	GCCTCTACACGGGACCCATA
	Probe	27477–27502	/6-FAM/CTCTTCACATAATCGCCCCGAGCTCG/BHQ1/
***N2***^**b**^	Forward primer	29424–29442	GGCACTGAGGACCCACGTT
	Reverse primer	29498–29477	TTGCGACATACCCATAAAAGCA
	Probe	29445–29471	/6-FAM/CCCCAAATTGCTGAGCTTGCTCCTACA/BHQ1/

a Primer/probe sequences were based on a report by Corman et al. [11].

b Primer/probe sequences were derived from a report by Lu et al. [12].

All genomic locations were based on the human betacoronavirus 2c EMC/2012 strain (GenBank: JX869059.2).

### Performance of the RT-RPA-VF assays with viral RNA spiked respiratory swabs RNA samples

Various concentrations of the MERS-CoV RNA were separately spiked in thirty respiratory swabs RNA extracted from throat, oral or nasal swabs at a volume ratio of 9:1 (the viral final concentrations ranged from 1.2 × 10^0^ to 1.2 × 10^6^ copies/μl) and used as experimental groups. Total RNA extracted from swab samples that was not spiked with the MERS-CoV RNA was used as a control group.

Two RT-qPCR assays for *UpE* and *N* were used to screen samples from the experimental and control groups, because two assays were recommended as the gold standard according to the WHO guidelines for laboratory testing for MERS-CoV [11,12]. Then, all samples were used to evaluate the RT-RPA-VF assay panel. Three replicates were analyzed in each trial.

## Results

### Primer and probe design

The conserved sequences of the *UpE* and *N* genes of the EMC strain were used as templates to design primers and probes. As shown in Table 3, for the *UpE* gene, the sense primer *UpE*-F (5’-TTAGCCTAGTTTCTGTAACTGACTTCTCCT-3’), reverse primer *UpE*-R (5’-Biotin-GTTCGTTTTCCATATGTCCAAAGAGAGAC-3’), and probe *UpE*-P (5’-FITC-GCGATTCAGTTCCTCTTCACATAATCGCCCCGAGC [THF] CGCTTATCG-C3 Spacer-3’) were designed to target a 193 bp section. For the *N* gene, the sense primer *N*-F (5’-Biotin-CCCTTGAGTCTGGCAAAGTAAAGCAATCGC -3’), reverse primer *N*-R (5’-AGGAGCAAGCTCAGCAATTTGGGGCCAACGTG-3’) and probe *N*-P (5’-FITC-AGCAATTTGGGGCCAACGTGGGTCCTCAGT[THF]CCGAGTTTATTCAAT-C3 Spacer-3’) were designed to target a 233 bp section. The probes were labeled with FITC at the 5′ end, THF in the internal region, and a blocking C3-spacer at the 3′ end. The reverse primer for the *UpE* gene and the forward primer of the *N* gene were labeled with biotin at the 5′ end.

**Table 3:** Sequences of the primers and probes used for the MERS-CoV RT-RPA-VF assay panel.

Primer Name	Primer Position	Sequence (5′ – 3′)
***UpE*-F**^**a**^	27396–27425	TTAGCCTAGTTTCTGTAACTGACTTCTCCT
***UpE*-R**^**b**^	27588–27560	Biotin-GTTCGTTTTCCATATGTCCAAAGAGAGAC
***UpE*-P**^**c**^	27466–27509	FITC-GCGATTCAGTTCCTCTTCACATAATCGCCCCGAGC(THF)CGCTTATCG-C3 Spacer
***N*-F**^**a**^	29236–29265	Biotin-CCCTTGAGTCTGGCAAAGTAAAGCAATCGC
***N*-R**^**b**^	29468–29437	AGGAGCAAGCTCAGCAATTTGGGGCCAACGTG
***N*-P**	29456–29411	FITC-AGCAATTTGGGGCCAACGTGGGTCCTCAGT (THF)CCGAGTTTATTCAAT- C3 Spacer

a forward primer

b reverse primer

c probe

All genomic locations were based on the human betacoronavirus 2c EMC/2012 strain (GenBank: JX869059.2).

### Optimizing the RT-RPA-VF reaction conditions

The reaction conditions of the RT-RPA-VF assays were optimized to improve sensitivity. First, 10-fold dilutions of synthesized RNA transcripts were used as a template for the RT-RPA-VF assays, and the reaction process was performed at different temperatures (42°C, 40°C, 39°C, 37°C, 35°C, 33°C, and 30°C) for 30 min. Table 4 shows a 100% positive rate at 33°C for the *UpE* assay and at 35°C for the *N* assay. Therefore, 33°C and 35°C were deemed the optimal temperatures for the RT-RPA-VF assays to detect the *UpE* and *N* genes, respectively.

**Table 4:** Optimization of the reaction temperature for RT-RPA-VF assays targeting the MERS-CoV *UpE* and *N* genes.

Temperature (°C)	Synthesized *UpE*-RNA transcript dilution (2.6×copies/μl)					Synthesized *N*-RNA transcript dilution (2.6×copies/μl)				
	10^3^	10^2^	10^1^	10^0^	Negative	10^3^	10^2^	10^1^	10^0^	Negative
**42**	3/3	3/3	0/3	0/3	0/3	3/3	1/3	0/3	0/3	0/3
**40**	3/3	3/3	0/3	0/3	0/3	3/3	3/3	0/3	0/3	0/3
**39**	3/3	3/3	0/3	0/3	0/3	3/3	3/3	0/3	0/3	0/3
**37**	3/3	3/3	0/3	0/3	0/3	3/3	3/3	1/3	0/3	0/3
**35**	3/3	3/3	1/3	0/3	0/3	3/3	3/3	3/3	0/3	0/3
**33**	3/3	3/3	3/3	1/3	0/3	3/3	3/3	0/3	0/3	0/3
**30**	3/3	3/3	1/3	0/3	0/3	3/3	0/3	0/3	0/3	0/3

The data were obtained from three independent experiments, and three parallels were analyzed in each experiment.

At the optimal reaction temperatures described above, 10-fold dilutions of the synthesized RNA transcripts were used as the templates to evaluate the amplification efficiency at different times (10 min, 15 min, 20 min, 25 min, and 30 min). According to the highest dilution at which 100% of replicates were positive, the optimal reaction time for the two assays was 25 min, as shown in Table 5.

**Table 5:** Optimization of the reaction times for RT-RPA-VF assays targeting the MERS-CoV *UpE* and *N* genes.

Times(min)	Synthesized *UpE*-RNA transcript dilution (2.6×copies/μl)					Synthesized *N*-RNA transcript dilution (2.6×copies/μl)				
	10^3^	10^2^	10^1^	10^0^	Negative	10^3^	10^2^	10^1^	10^0^	Negative
**10**	0/3	0/3	0/3	0/3	0/3	0/3	0/3	0/3	0/3	0/3
**15**	0/3	0/3	0/3	0/3	0/3	1/3	0/3	0/3	0/3	0/3
**20**	3/3	0/3	0/3	0/3	0/3	3/3	3/3	0/3	0/3	0/3
**25**	3/3	3/3	3/3	1/3	0/3	3/3	3/3	3/3	3/3	0/3
**30**	3/3	3/3	3/3	1/3	0/3	3/3	3/3	3/3	3/3	0/3

The data were obtained from three independent experiments, and three parallels were analyzed in each experiment.

### Quantification of the MERS-CoV RNA

**Fig 3:**
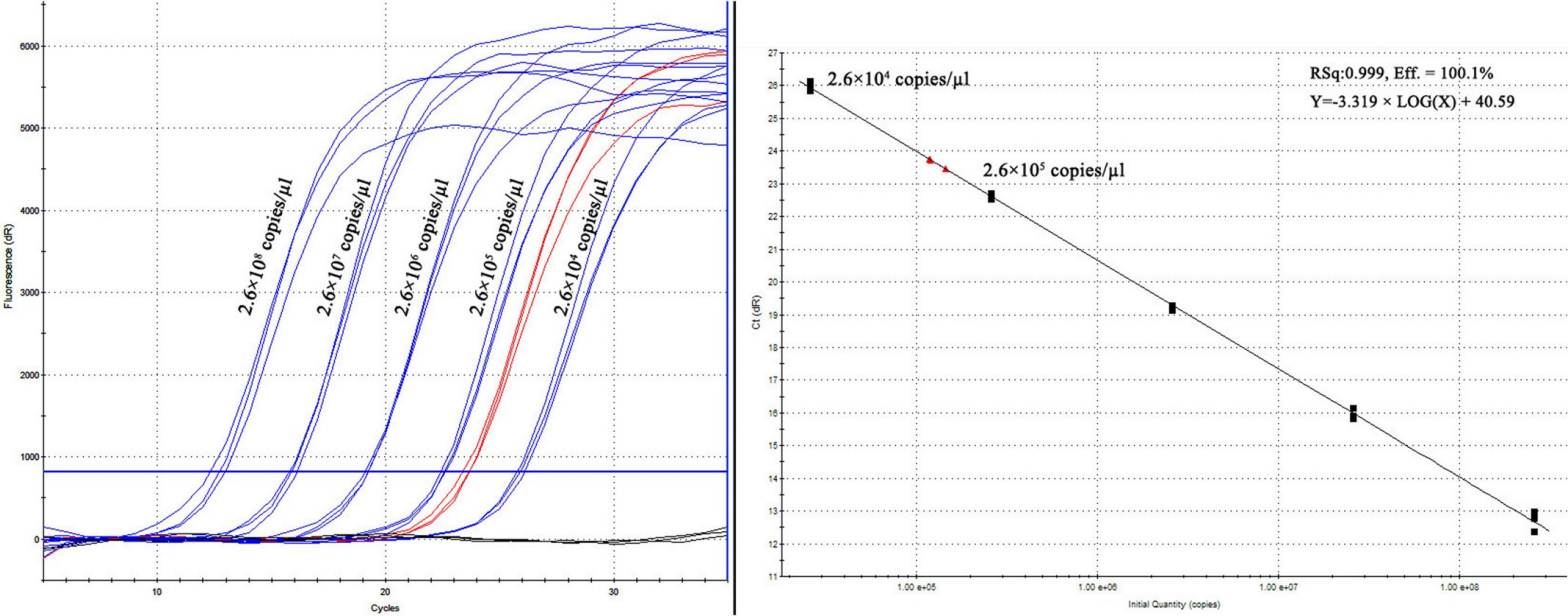
Quantitative analysis of the MERS-CoV RNA using the absolute RT-qPCR assay targeting *UpE* gene. Ten-fold dilutions of synthesized *UpE* RNA transcripts were used to establish the standard curve, and 100-fold dilutions of total RNA from virus-infected cell suspensions were used as the test template.

An absolute RT-qPCR assay was performed using primer and probe sequences against the *UpE* gene described in a previous report to quantify the concentration of the MERS-CoV RNA in a total RNA sample containing 5 × 10^6^ PFU/ml virus in the culture [11]. Ten-fold dilutions of synthesized *UpE*-RNA transcripts were used to establish the standard curve, 100-fold dilutions of total RNA from virus-infected cell suspensions were used as the test template, and the RNA of uninfected cell suspensions was used as the negative control template. Linear amplification was achieved from 2.6 × 10^4^ to 2.6 × 10^8^ copies/μl, with a calculated linear correlation coefficient (*R*2) of 0.999 and efficiency value of 100.1% (Fig 3). Next, the concentration of the MERS-CoV RNA was calculated as 1.2 × 10^9^ copies/μl in 461 ng/μl total RNA or 5 × 10^6^ PFU/ml virus in using a positive correlation between cycles and concentration. The known concentration of viral RNA was used to evaluate the RT-RPA-VF assays.

### Analytical sensitivity and specificity

Ten-fold serial dilutions of RNA transcripts were detected in 24 replicates to evaluate the sensitivity of the RT-RPA-VF assays and 100% of replicates were positive. As shown in Table 6, the *UpE* assay was capable of detecting 2.6 × 10^1^copies/μl and the *N* assay detected at least 2.6 copies/μl RNA transcripts.

**Table 6:** Limits of detection of the MERS-CoV RT-RPA-VF assay panel using RNA transcripts.

RNA transcript (copies/μl)	Positive/Tested (%)	
	*UpE* assays	*N* assays
**2.6 × 10**^**6**^	24/24 (100%)	24/24 (100%)
**2.6 × 10**^**5**^	24/24 (100%)	24/24 (100%)
**2.6 × 10**^**4**^	24/24 (100%)	24/24 (100%)
**2.6 × 10**^**3**^	24/24 (100%)	24/24 (100%)
**2.6 × 10**^**2**^	24/24 (100%)	24/24 (100%)
**2.6 × 10**^**1**^	24/24 (100%)	24/24 (100%)
**2.6 × 10**^**0**^	7/24 (29%)	24/24 (100%)
**2.6 × 10**^**−1**^	0/24 (0)	1/24 (4%)

**Fig 4:**
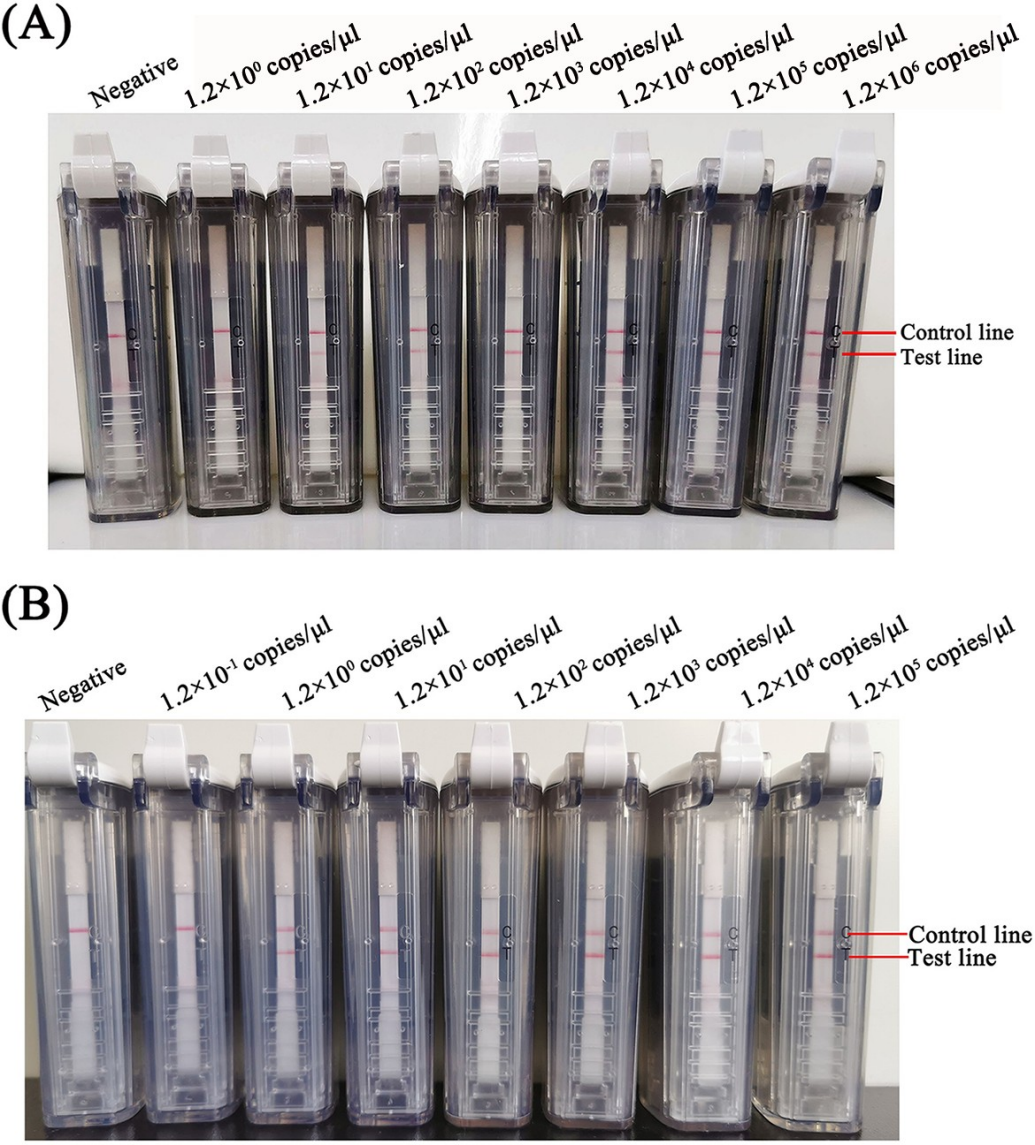
Detection of 10-fold serial dilutions of the MERS-CoV RNA using the RT-RPA-VF assays against the *UpE* (A) and *N* (B) genes.

Ten-fold dilutions of viral RNA were used as templates for the two RT-RPA-VF assays under the same reaction conditions to further validate the sensitivity. The limit of detection of the *UpE* assay was 1.2 × 10^1^ copies/μl, and the limit of detection for the *N* assay was 1.2 copies/μl viral RNA (Fig 4A and 4B).

**Fig 5:**
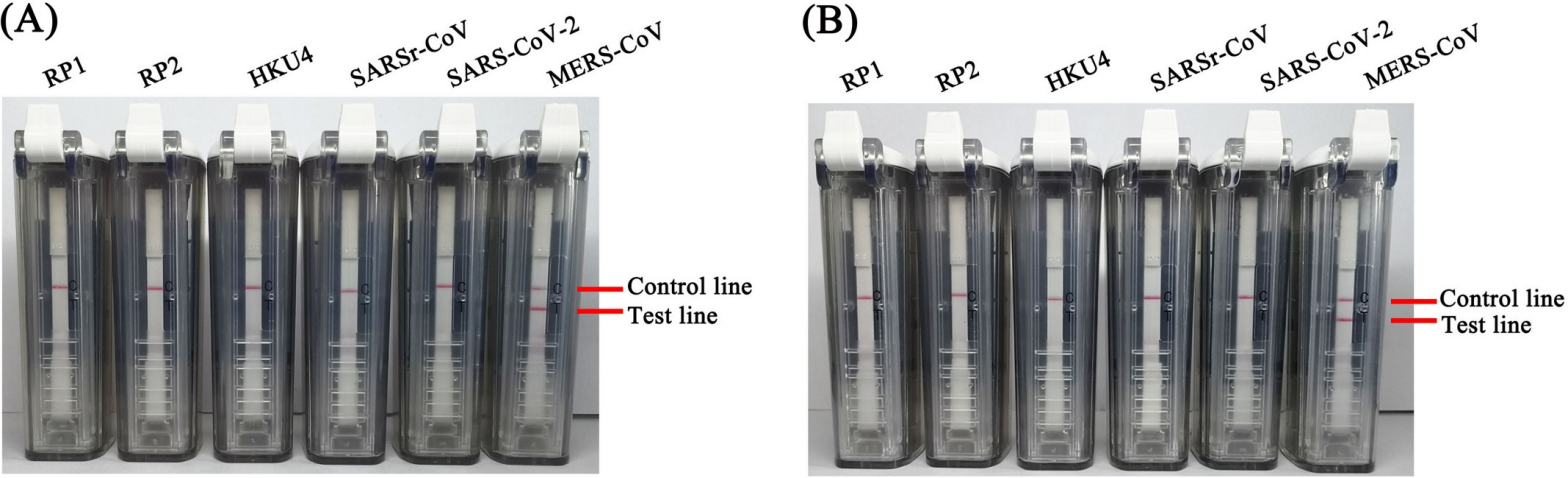
**The specificity of the RT-RPA-VF assays against the *UpE* (A) and *N* (B) genes.** RP1 controls and RP2 controls kits are panels of nucleic acids from multiple respiratory pathogens, including 229E, OC43, NL63, HKU1, influenza A/B, rhinovirus, adenovirus, and parainfluenza.

Nucleic acids from a respiratory pathogen panel and other coronaviruses were detected to verify the specificity of the MERS-CoV RT-RPA-VF assays. The results showed that only the MERS-CoV RNA was positive. Therefore, no false-positive test results were obtained for the MERS-CoV RT-RPA-VF assays using nucleic acids obtained from 229E, OC43, NL63, HKU1, influenza A/B, SARSr-CoV, HKU4, SARS-CoV-2, etc. (Fig 5).

### MERS-CoV RNA was spiked in extracted RNA samples to evaluate the RT-RPA-VF assays

Two gold standard RT-qPCR assays were used to determine whether all RNA-spiked samples in experimental groups were positive and all unspiked samples in control groups were negative. Then, these samples were detected using the RT-RPA-VF assay panel. This test showed that the RT-RPA-VF assay for the *UpE* gene had a sensitivity of 86% (95% confidence interval [CI], 0.80–0.90) and a specificity of 100% (95% CI, 0.89–1.00). The RT-RPA-VF assay for the *N* gene had a sensitivity of 100% (95% CI, 0.98–1.00) and a specificity of 100% (95% CI, 0.89–1.00). When we compared the gold standard with our assay panel, two gold standard RT-qPCR assays detected concentrations as low as 1.2 copies/μl viral RNA, and these results were consistent with previous reports [12]. The sensitivity of the RT-RPA-VF assay for the MERS-CoV *UpE* gene was 1.2 × 10^1^ copies/μl viral RNA, which was lower than RT-qPCR for the *UpE* gene (Fig 4A). The coincidence rate between RT-RPA-VF and RT-qPCR for the *UpE* gene at 87.5% (kappa = 0.6). The limit of detection of the RT-RPA-VF assay targeting the *N* gene was 1.2 copies/μl viral RNA, with a sensitivity equivalent to the RT-qPCR assay (Fig 4B). The coincidence rate between the RT-RPA-VF assay against the *N* gene and gold standard was 100% (kappa = 1), indicating the two assays displayed high consistency (Table 7).

**Table 7:** The sensitivity and specificity of the RT-RPA-VF assay panel were evaluated using spiked extracted RNA specimens.

Target	The RT-RPA-VF assay panel	Two gold standard RT-qPCR assays		Sensitivity	Specificity	Concordance rate (%)
		Positive samples^a^(N = 210)	Negative samples^b^(N = 30)	Estimated value (95% CI)^c^		
***UpE* gene**	Positive	180	0	86% (0.80–0.90)	100% (0.89–1.0)	87.5%
	Negative	30	30			
***N* gene**	Positive	210	0	100% (0.98–1.0)	100% (0.89–1.0)	100%
	Negative	0	30			

a Positive samples were 10-fold dilutions of viral RNA ranging from 1.2 × 10^1^ to 1.2 × 10^7^ copies/μl spiked in RNA specimens extracted from respiratory swabs at a volume ratio of 9:1, and all spiked extracted RNA specimens were confirmed by gold standard RT-qPCR assays.

b Negative samples were RNA purified from uninfected Huh7 cell suspensions at different time points mixed with RNA specimens extracted from respiratory swabs.

c 95% CI: The 95% confidence interval.

## Discussion

In recent years, epidemics caused by newly emergent viruses such as SARS, MERS, Ebola virus disease, Zika disease and COVID-19 have occurred more frequently [24–27]. More remarkably, SARS-CoV-2 has infected more than six million people in the last six months. Considering recent experience related to the prevention and control of COVID-19, a rapid diagnostic assay is important in the initial emergency response stage of an epidemic to reduce the workload of laboratory technicians, reduce the risk of cross-infection and ensure the timely treatment of patients. Therefore, we referenced the needs of SARS-CoV-2 diagnosis and designed a rapid and convenient RT-RPA-VF assay for detecting MERS-CoV.

A variety of MERS-CoV detection methods have been established, including nucleic acid amplification, antigen detection, and serological detection. Compared with antigen detection methods, such as enzyme-linked immunosorbent assays and colloidal gold immunochromatographic assay, nucleic acid amplification detection methods have the disadvantages of requiring professional technical operators and sophisticated equipment, but they have higher sensitivity and accuracy. Serological tests display cross-reactivity with SARS-CoV and are mostly used for a retrospective diagnosis [28]. Notably, the RT-RPA-VF assay is a promising approach for the differential diagnosis of emerging or re-emerging infectious diseases, as it combines the advantages of convenient antigen detection with the good sensitivity of nucleic acid detection.

In this study, we describe the RT-RPA-VF assay panel targeting the MERS-CoV *UpE* and *N* genes. Compared to other MERS-CoV molecular detection methods, such as PCR, nested PCR, RT-qPCR, and the one-pot RT-LAMP assay, the RT-RPA-VF assay provides a shorter reaction time at a lower temperature [12,29,30]. Early in the emergence of MERS-CoV, Abd El et al. developed a real-time RT-RPA method to target the *ORF1b* gene that detected 10 RNA molecules of MERS-CoV [31]. The RT-RPA-VF assay reported in our study has a sensitivity equal to the real-time RT-RPA method reported by Abd El. Notably, the RT-RPA-VF assay maybe more appropriate as a rapid and mobile molecular MERS-CoV monitoring assay because no fluorescence signal capture equipment is required. This assay only requires a closed disposable vertical flow visualization strip (VF) device that is similar in size to a half palm-sized box. Moreover, aerosol contamination of basic RPA assays can be solved by a closed VF.

Although the RT-RPA-VF assay has some advantages, the limitations of RT-RPA-VF assay was that not suitable the large size of samples, as the operator must individually place the RT-RPA reaction tube into the disposable VF device after isothermal amplification. Additionally, its primers and probe are relatively complicated. RPA primers and probe can tolerate 5–9 mismatched bases [21]. If only a couple of primers are used, a high level of incorrect amplification may occur. The introduction of the probe and its THF site may relatively improve the specificity of amplification. Currently, no professional software is available for designing RPA primers and probes. Effective primer and probe only are identified through a stringent screen. The reaction conditions must be optimized for effective primers and probes to achieve high sensitivity. Based on our data, the optimal temperatures for the *UpE* and *N* assays in the same reaction solution were 33°C and 35°C, respectively. Thus, the four enzymes of RT-RPA have strong activity within a certain temperature range, but the binding and extension of the primers and probe to the template must occur at a specific temperature to maximize their effectiveness. Additionally, an appropriate reaction time is significance for improve sensitivity.

The mean viral load in the lower respiratory tract of MERS-CoV infected patients is approximately 5.01 × 10^6^ copies/ml [32]. The viral load in upper respiratory tract specimens may range from 10^3^ to 10^6^ copies/ml during an acute infection [33]. In our assay panel, the limit of detection of the viral RNA was 1.2 × 10^1^ copies/μl for the *UpE* assay and 1.2 copies/μl for the *N* assay in 30 min. Therefore, the sensitivity of each RT-RPA-VF assay was sufficient for a MERS-CoV diagnosis. However, we still chose to retain two different targets in MERS-CoV for specimen testing according to WHO guidelines, in which RT-qPCR assays for the *UpE* gene are used for screening and assays for the *ORF1a*, *ORF1b* or *N* genes are used for confirmation to prevent misidentification of MERS-CoV cases. According to our data, the RT-RPA-VF assay for the *N* gene exhibited higher sensitivity than the *UpE* assay, and thus we recommend the *N* assay for screening and the *UpE* assay for diagnosis. Compared with gold standard RT-qPCR assays, the sensitivity of the RT-RPA-VF assay for the *UpE* gene was less than the 1.2 copies/μl detection limit of the RT-qPCR for the *UpE* gene. However, the RT-RPA-VF assay targeting the *N* gene detected as low as 1.2 copies/μl viral RNA, with a sensitivity equivalent to the RT-qPCR assay for the *N* gene. Based on these results, the RT-RPA-VF assay panel has the potential to be used as an alternative to RT-qPCR for MERS-CoV.

MERS-CoV is a zoonotic pathogen, and the dromedary camel is the main host for transmission to humans. People who work in camel farms, slaughterhouses and markets are at risk, and infected people may spread the virus to their contacts. Because dromedary camels have a large geographical range, the disease is subclinical and camels are significant animal products, vaccination and culling to block animal transmission are difficult to implement [34–36]. Rapid detection is essential for prospective MERS-CoV surveillance. Therefore, the RT-RPA-VF assay panel was originally designed to detect all MERS-CoV strains from humans and dromedary camels. As China is a nonepidemic country of MERS-CoV, we were unable to obtain clinical specimens, but spiked respiratory swabs RNA were used to evaluate RT-RPA-VF assays and determine the clinical feasibility. Given that DPP4 is the receptor for MERS-CoV, and it is mainly distributed in the lower respiratory tract in humans and the upper respiratory tract in camels; thus, different types of samples are collected from humans and camels. Therefore, throat swabs were collected from healthy humans, and oronasal swabs were collected from Camelidae (camels and alpacas). The MERS-CoV RNA was spiked in the total RNA extracted from the swabs to evaluate the assay panel. The results showed that swab RNA did not affect the limit detection and specificity of the RT-RPA-VF assays. However, we have not conclusively confirmed that this assay panel can be used to diagnose MERS because it has not been evaluated using clinical samples. Therefore, we are eager to enlist researchers to help us validate this assay in the future.

On the other hand, the two RT-RPA-VF assays targeting the MERS-CoV *UpE* and *N* genes were rapid (within 30 min from amplification to observation) and highly sensitive (as little as 1.2–1.2 × 10^1^ copies/μl viral RNA), and no sophisticated equipment was needed. Thus, this assay is promising for the detection and active surveillance of MERS-CoV in low-resource areas. Furthermore, this assay can minimize risk of false positives and the requirement to ship specimens. The design of the RT-RPA-VF assay can also be used to develop methods for the rapid detection of other infectious diseases. According to previous reports, fluid samples can be quickly heated directly to release RNA without RNA purification and used in the RT-RPA assay [37,38]. We speculate that this assay will be combined with quickly handled fluid samples without RNA extraction, showing potential for use in point-of-care testing for highly contagious tropical diseases. Certainly, this assumption still requires verification using MERS-CoV infected clinical samples.
